# Longitudinal Associations between Parental Support and Parental Knowledge on Behavioral and Emotional Problems in Adolescents

**DOI:** 10.1007/s10964-021-01559-0

**Published:** 2021-12-23

**Authors:** Olalla Cutrín, Lorena Maneiro, Yasmynn Chowdhury, Stephen S. Kulis, Flavio F. Marsiglia, José Antonio Gómez Fraguela

**Affiliations:** 1 Universidade de Santiago de Compostela, Santiago de Compostela, Spain; 2 Leiden University, Leiden, The Netherlands; 3 University of Oxford, Oxford, UK; 4 Arizona State University, Phoenix, AZ USA

**Keywords:** Parenting practices, Adolescent adjustment, Mediation effects, Prevention

## Abstract

When testing longitudinal effects of parenting practices on adolescent adjustment, an integrated consideration of externalizing and internalizing behaviors is a gap in research. This study analyzed how parental support and parental knowledge directly and indirectly influence both antisocial behavior and emotional problems. The sample had 642 adolescents aged 12-15 (mean age = 12.49; 45.4% females) from Spain, who participated in a three-year long study. The results showed longitudinal bidirectional associations between parental support and parental knowledge. Only parental knowledge, however, directly predicted antisocial behavior and emotional problems. Parental support had an indirect effect on outcomes through the mediating effect of parental knowledge. This study has practical implications by indicating that increasing parental knowledge should be the target of educational-prevention programs.

## Introduction

Research has demonstrated consistently that negative parenting practices are a robust risk factor for antisocial behavior and delinquency (Hoeve et al., 2009), as well as emotional problems in adolescence (Garthe et al., 2015). Both antisocial behavior and emotional problems are quite common during adolescence. The most recent findings from the Health Behavior in School-aged Children study in Spain indicated that 13.2% of adolescents committed shoplifting, 8.8% were involved in acts of vandalism, and 16% had been involved in a violent dispute with a teacher (Moreno et al., 2019). Among Spanish youth aged 15 to 24 years old, prevalence rates for depression and anxiety were 1.22% to 2.06%, respectively (Ministerio de Sanidad, Consumo y Bienestar Social & Instituto Nacional de Estadística, 2017). The co-occurrence of externalizing and internalizing problems during adolescence is well-established (Loeber & Burke, 2011), as are the negative consequences of antisocial behavior (DeLisi, 2016) and mental health problems (Hughes & Gullone, 2008) for the psychological, social and economic well-being of youth, their families, and their broader communities. Despite the high co-occurrence of these issues and the relevance of parenting practices in adolescent adjustment, there is a gap in research on how the interrelation of parenting practices during adolescence influences both behavioral and emotional problems. This work seeks to fill gaps in the extant literature by analyzing longitudinal mediation effects of parental support and parental knowledge on both antisocial behavior and emotional problems in Spanish adolescents.

### Parental Knowledge as a Predictor of Antisocial Behavior and Emotional Adjustment

Parental knowledge (i.e., information that parents have about their child’s activities, whereabouts, or friendships) is one of the most robust factors predicting adolescent antisocial behavior (Kerr & Stattin, 2000; Stattin & Kerr, 2000). Research has demonstrated that the level of parental knowledge, rather than a supervision strategy involving active monitoring, is linked to adolescent behavior (Eaton et al., 2009). In this regard, adolescent willingness to disclose information to parents is a strong predictor of higher levels of parental knowledge and lower levels of antisocial behavior (Kerr et al., 2010). There is enduring evidence of a strong association between lower levels of parental knowledge and higher levels of adolescent antisocial behavior, both violent and non-violent, as well as substance use behaviors (e.g., Cutrín et al., 2019). Similarly, levels of parental knowledge are negatively correlated with and/or predictive of occurrences of emotional problems, such as symptoms of anxiety and depression (Bacchini et al., 2011), with more consistent evidence for the effects of parental knowledge on depression than on anxiety (Yap et al., 2014).

### Parental Support as a Predictor of Antisocial Behavior and Emotional Adjustment

A supportive parental style, based on acceptance, affection, attachment, warmth, communication, and intimacy, has been demonstrated generally to be a protective factor against antisocial behavior (Hoeve et al., 2009) and substance use (Calafat et al., 2014), and is associated with better adolescent adjustment. Overall, lower levels of parental support have been associated with higher levels of antisocial behavior in adolescence (Álvarez-García et al., 2016), drug use and externalizing behavior (Parra & Oliva, 2006). Likewise, lower levels of perceived parental support and lack of parental displays of warmth are associated with a higher risk of developing depressive symptoms and anxiety in adolescence (Yap et al., 2014), and parental support is more strongly associated with depressive symptoms than with anxiety symptoms (Pinquart, 2017).

### Mediation Parental Support-Knowledge Influencing Adolescent Behavior and Adjustment

From a developmental theoretical perspective, which is intrinsically a holistic-integrative approach, the risk factors promoting maladjustment in adolescence interact with each other at early stages (Compas, 2004). Adolescent behavior is viewed as the result of a bioecological process in which different risk factors are linked to one another depending on the interaction of different systems and contexts (Bronfenbrenner, 2005). Family is one of the most important systems that affects adolescent development (Magnusson & Stattin, 2006). Within the family system, developmental theory showed that the interrelation between multiple negative parenting practices works as a risk factor for developing maladjusted outcomes (Granic et al., 2003).

As components of the same system, positive parent-adolescent relationships and parental knowledge are linked in a bi-directional way (Walters, 2019). However, parent-adolescent communication processes have been evaluated most often as predictors of disclosure and knowledge (Laird & Zeringue, 2019), and not the reverse way. From a developmental perspective, parent-adolescent relationships are shaped by previous patterns of family interactions (Collins & Steinberg, 2008), such that the establishment of a supportive and communicative family atmosphere during childhood promotes adolescent disclosure and parental knowledge during adolescence (Kerr et al., 2010). Positive open communication, closeness, and supportive family bonds facilitate parenting practices involving supervision and knowledge (Fletcher et al., 2004). As has been found in other studies, parent-child relationships involving warmth (Blodgett Salafia et al., 2009) and perceived support (Tilton-Weaver, 2014) promote adolescents’ willingness to spontaneously disclose information about their lives, and such disclosure is the critical source and main predictor of parental knowledge (Stattin & Kerr, 2000). Therefore, from a developmental perspective, warm and supportive parent-child relationships are the prerequisite for child disclosure and, ultimately, parental knowledge (Liu et al., 2020).

Previous studies have suggested that parental support does not even exert a direct effect on antisocial behavior in adolescence (e.g., Cutrín et al., 2015; de Kemp et al., 2007), but supportive parent-child relationships do influence such behaviors through the degree of parental supervision or parental knowledge (Burfeind & Bartusch, 2016). Research has consistently found that higher levels of parental support are indirectly related to lower levels of antisocial behavior (Yun et al., 2016) and lower levels of antisocial beliefs (Dane et al., 2012) through the mediating role of parental knowledge. Parental support appears to be indirectly and inversely associated with antisocial behavior and substance use through the mediation of parental knowledge among both the general adolescent population and juvenile offenders (Cutrín et al., 2017). However, this study had a cross-sectional design, and it could not confirm any hypotheses regarding directionality in the parenting variables.

Despite the vast body of research on externalizing problems and antisocial behavior, there is limited research on the mediation effects of parental support through parental knowledge on internalizing and emotional problems in adolescence. A study on the effects of parental support on depressive symptoms in children and pre-adolescents has analyzed mediation effects of parent-child attachment measured, in part, as open parent-child communication (Yan et al., 2017). That study found that children whose parents support their autonomy showed fewer depressive symptoms by establishing a more secure attachment. To the best of our knowledge, there is no prior research on how parental knowledge mediates the effects of parental support on internalizing problems, as well as on both behavioral and emotional problems in adolescence.

From a developmental and holistic-integrative approach, the study of the longitudinal interrelation of the main positive parenting practices influencing co-occurring behavioral and emotional problems is warranted to better understand adolescent development. In addition, although developmental theory suggests a specific direction for the effects of parental support and parental knowledge on adolescent adjustment, none prior research has empirically confirmed this finding by testing the mediation effects of both parenting practices. By addressing this gap around considering an integrative approach on parenting practices and adolescent adjustment, and the need for longitudinal designs, this study will contribute knowledge on the effects of parental support and parental knowledge on both externalizing and internalizing problems and may contribute to the development of prevention strategies, at both behavioral and emotional levels, for adolescents and their parents.

## The Current Study

To address gaps in the extant literature, the current study analyzed bi-directional relationships and longitudinal mediation effects of parental support and parental knowledge on antisocial behavior (i.e., aggression, rule breaking, and drug-use problems) and emotional problems in a general (school-based) population of Spanish adolescents. Three main hypotheses guided the current study from a holistic and developmental perspective. Bi-directional positive effects between parental support and parental knowledge were expected; that is, higher levels of parental support would predict higher levels of parental knowledge, and higher levels of parental knowledge would predict higher levels of parental support one year later (Hypothesis 1). As robust predictors for externalizing and internalizing problems, it was also expected that parental support and parental knowledge would negatively predict antisocial behavior and emotional problems (Hypothesis 2). Finally, because parental support and knowledge were expected to be bi-directionally related, potential mediation effects were tested in both directions. However, based on developmental theory and previous research, only a unidirectional pathway was expected to be significant: that parental support indirectly influences adolescent adjustment through the mediating role of parental knowledge (Hypothesis 3).

## Methods

### Sample

The current study engaged a sample of students who participated in a three-year (2017–2019) longitudinal study across 11 public secondary schools in Galicia (NW Spain). At baseline (T1), the sample included 642 adolescents enrolled in the first grade of Spanish compulsory secondary education [1^st^ grade ESO; equivalent to 7^th^ grade in the US]. Adolescents ranged in age from 12 to 15 (*M* = 12.49; *SD* = 0.67; 92% were age typical for their grade level, 12-13 years old) and 45.4% were female. The second wave of the data collection (T2) took place approximately 12 months after the initial assessment and included 493 adolescents aged 13-16 (*M* = 13.36; *SD* = 0.59), 48.3% females. At T2, 99.6% of adolescents were enrolled in the second grade of compulsory secondary education [2^nd^ grade ESO] and 0.4% were repeating 1^st^ grade ESO. The third wave (T3), which was carried out two years after the initial assessment, included 414 participants aged 14-16 (*M* = 14.18; *SD* = 0.40), 49.0% females, of which 392 were followed up from both T1 and T2. At T3, 97.8% of adolescents were enrolled in the third grade of compulsory secondary education [3^rd^ grade ESO] and 2.2% were repeating 2^nd^ grade ESO. At the beginning of the study, the majority of participants lived with both parents (78.3%), whereas 16.8% lived only with their mother, 2.3% lived only with their father, and 2.5% lived with other relatives. More than 90% of the sample was white, born in Galicia, and came from middle and low-middle socio-economic backgrounds.

The level of attrition was 23.2% between T1 and T2 and 38.9% between T1 and T3. Significant differences were found between respondents and non-respondents at T3 with regard to baseline gender χ^2^(1) = 6.19, *p* = 0.013, age *t*(639) = 9.40, *p* < 0.001, antisocial behavior *F*(1, 578) = 56.455, *p* < 0.001, and emotional problems *F*(1, 615) = 11.221, *p* = 0.001. Non-respondents at T3 were more likely to be male, older, and reporting higher levels of antisocial behavior and emotional problems at T1 than respondents at follow-up.

### Measurements

#### Parental knowledge (T1-T2)

The degree of parental knowledge regarding adolescents’ whereabouts, activities, and friendships was measured by a self-reported scale composed of 8 items (Kerr & Stattin, 2000; Stattin & Kerr, 2000) and validated in community Spanish adolescents (Cutrín et al., 2019) (e.g., “Your parents know what you do during your free time”). Items were scored from 0 (*never*) to 3 (*always*), higher scores indicating higher levels of knowledge. This scale presented an internal consistency of α = 0.81 in T1 and α = 0.80 in T2.

#### Parental support (T1-T2)

The perception of parental warmth, responsiveness, and closeness to parents was assessed by means of a self-reported scale, validated in community Spanish adolescents (Oliva et al., 2007). The scale was composed of 8 items (e.g., “You feel supported and understood”) scored in a 4-point scale ranging from 0 (*never*) to 3 (*always*), higher scores indicating higher levels of support. The internal consistency of the scale was α = 0.90 in T1 and α = 0.91 in T2.

#### Antisocial behavior (T1-T3)

Three self-reported scales from the short Spanish version of the Antisocial Behavior Questionnaire (ABQ; Luengo et al., 1999) were used to evaluate *aggression* (6 items; α = 0.72; e.g., “Fighting and hitting someone”), *rule-breaking* (6 items; α = 0.63; e.g., “Spending the night out without permission”), and *drug-related problems*, that is, unhealthy and antisocial behaviors derived from substance abuse (6 items; α = 0.63; e.g., “Having five or more drinks on one occasion”). Items were scored from 0 (*never*) to 3 (*very often*), higher scores indicating higher levels of antisocial behavior. The composite from these three scales showed an internal consistency of α = 0.81 in T1 and α = 0.64 in T3.

#### Emotional problems (T1-T3)

The self-reported scale on emotional symptoms from the Spanish version of the Strengths and Difficulties Questionnaire (SDQ; Ortuño-Sierra et al., 2015) was used to assess the presence of nervousness, sadness, or worry in adolescents (e.g., “I often feel sad or discouraged or feel like crying”). This scale was composed of 5 items scored from 0 (*it’s not true*) to 2 (*absolutely true*), higher scores indicating higher levels of emotional problems. The internal consistency of this scale was α = 0.71 in T1 and α = 0.73 in T3.

### Procedure

This study was approved by the Galician Autonomous Community’s Government and met ethical standards established by the Santiago de Compostela University’s bioethics committee. The investigators presented the study to the principals of 24 secondary schools, selected by convenience sampling. In the secondary schools that agreed to participate in the longitudinal study (11 schools), all students enrolled in the first grade (equivalent to 7^th^ grade in the US) were invited to participate and more than 90% of them completed the questionnaire.

Qualified psychologists from the research group visited the schools, explained the objectives of the research, and provided proper instructions to the respondents. Adolescents filled out the questionnaires in classroom sessions of approximately 50 minutes. Parents provided informed consent each year before the start of data collection according to the schools’ internal regulations. Passive consent was the most common approach, except in two schools that implemented active consent protocols. Parents received an informational note from their children, who had to return the signed consent to their teacher or school counselor. Subsequently, adolescents provided their assent before completing the questionnaire. Adolescent participation was voluntary, and their responses were anonymous and confidential. Three waves of data collection were implemented during a three-year period, with intervals of approximately 12 months between observations.

### Statistical Analyses

**Fig. 1:**
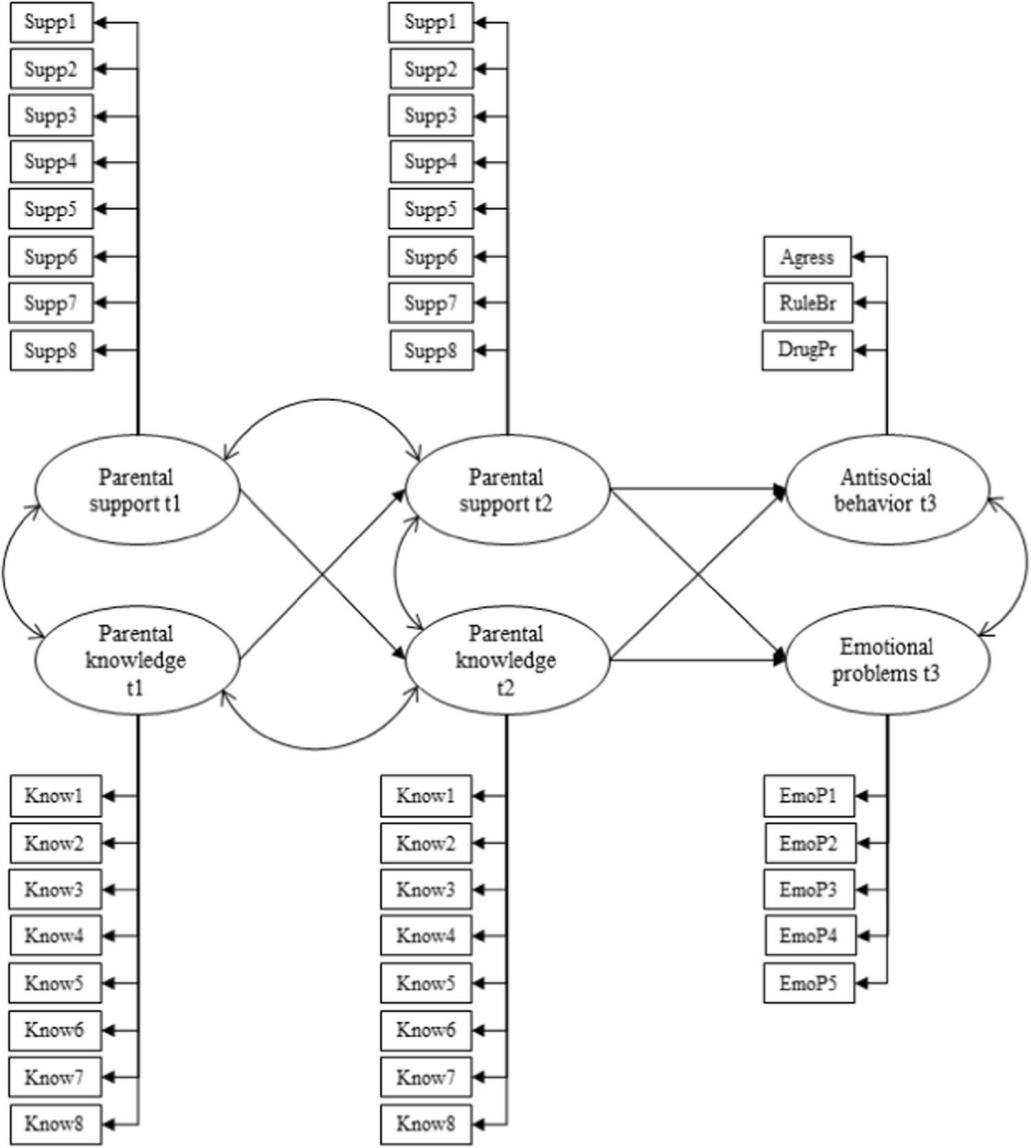
Proposed Model to Predict Antisocial Behavior and Emotional Problems. All the direct effects in the model controlled for gender and age in T1 (both observed variables). The direct effects on the outcomes also controlled for antisocial behavior and emotional problems in T1 (both observed variables). Factor loadings of support ranged from 0.61 to 0.81 in T1 and 0.69 to 0.81 in T2; factor loadings of knowledge ranged from 0.46 to 0.66 in T1 and 0.45 to 0.73 in T2; factors loadings of antisocial behavior ranged from 0.48 to 0.86 and factor loadings of emotional problems from 0.42 to 0.75; all of them being significant (*p* < 0.001)

Firstly, descriptive statistics and zero-order correlations among all the study variables (created by the mean of the items) were produced in IBM SPSS 25. The Bonferroni correction was applied to account for multiple comparisons. Next, structural equation modeling (SEM) was conducted in Mplus 7.4 using the robust maximum likelihood (MLR) estimator to adjust for non-normal distributions. SEM was used to test the effect of parenting practices (i.e., parental support and parental knowledge) on both antisocial behavior and emotional problems (see Fig. 1 for specific indicators of each latent variable). Parental support in T1 was included as a predictor of parental knowledge in T2 and parental knowledge in T1 as predictor of parental support in T2. Parental support and knowledge were set to intercorrelate at T1 as well as at T2. Intracorrelations between parental support at T1 and T2 and between parental knowledge at T1 and T2 were also specified in the model. Both parenting variables at T2 were included as predictors of antisocial behavior and emotional problems in T3. The model controlled for gender, age, antisocial behavior, and emotional problems in T1. Because these variables serve only for control purposes, they were included as observed variables in the model (antisocial behavior and emotional problems were measured as the mean of the component items) to conform as much as possible to the standards on the number of indicators that should be used in SEM (Bentler & Chou, 1987). The model also adjusted statistically for the school-level clustering of data to account for between-school variance and employed full information maximum likelihood (FIML) estimation to account for attrition to the posttests and item missing data.

Potential mediation effects of parental support as well as parental knowledge on both externalizing and internalizing problems were also tested in the model. A combination of Maximum Likelihood (ML) and bootstrapping (*b* = 5000) were used in order to maximize accurate estimations under a non-normal distribution and estimate bias-corrected 95% confidence intervals for indirect effects (Hancock & Liu, 2012). The following goodness-of-fit indexes were used to evaluate the model fit: χ^2^/*DF*, CFI, RMSEA, and SRMR, with χ^2^/*DF* < 2-3, CFI values of 0.95 or higher, and RMSEA and SRMR values lower or equal to 0.05 considered indicators of optimal model fit, whereas values of χ^2^/*DF* < 4, CFI > 0.90, and RMSEA and SRMR between 0.08 and 0.10 indicated adequate model fit (Hu & Bentler, 1999).

Finally, a sensitivity analysis was conducted to check the robustness of measurement and modeling. The model was analyzed using only rule-breaking as the antisocial behavior outcome to confirm whether the results can be replicated with a homogeneous measure of antisocial behavior, rather than the heterogeneous measure that also includes aggression, drug use and drug-related problems.

## Results

This study reports all the results of the tests of hypotheses, whether significant or not. Table 1 shows the descriptive statistics and intercorrelations among all the study variables. Both parenting practices had very high means, while antisocial behavior and emotional problems had low to very low means. After applying the Bonferroni correction, threshold levels of significance were set at 0.001 (45 comparisons). Correlations showed that females reported significantly higher levels of knowledge at T1 and emotional problems at T3 than males. Age was significantly and inversely associated with parental support at T1 and parental knowledge at T1 and T2, and positively associated with antisocial behavior and emotional problems at T1. Parental practices were significantly and positively related with each other. Specifically, both parental support and parental knowledge evidenced high stability over time, showing strong positive correlations between the first and second waves of data collected. Overall, both parenting practices were negatively associated with antisocial behavior and emotional problems after one-year and two-year follow-ups. Significant, positive correlations were found between antisocial behavior and emotional problems, except antisocial behavior at T3 with emotional problems at T1.

**Table 1:** Descriptive Statistics and Zero-Order Correlations Among the Main Study Variables

	1	2	3	4	5	6	7	8	9	10
1. Gender	1									
2. Age	−0.05	1								
3. Parental support T1	0.09	−0.21^***^	1							
4. Parental support T2	0.03	−0.07	0.53^***^	1						
5. Parental knowledge T1	0.14^***^	−0.26^***^	0.58^***^	0.36^***^	1					
6. Parental knowledge T2	0.03	−0.17^***^	0.38^***^	0.43^***^	0.62^***^	1				
7. Antisocial behavior T1	−0.13	0.31^***^	−0.43^***^	−0.22^***^	−0.56^***^	−0.38^***^	1			
8. Antisocial behavior T3	−0.12	0.02	−0.22^***^	−0.16^***^	−0.29^***^	−0.34^***^	0.32^***^	1		
9. Emotional problems T1	0.10	0.13^***^	−0.28^***^	−0.29^***^	−0.19^***^	−0.11	0.19^***^	0.12	1	
10. Emotional problems T3	0.32^***^	0.08	−0.17^***^	−0.25^***^	−0.17^***^	−0.20^***^	0.17^***^	0.27^***^	0.50^***^	1
*M (SD)*	–	12.49 (0.67)	18.90 (5.24)	18.52 (5.16)	19.15 (4.31)	18.20 (4.19)	0.77 (1.60)	0.68 (1.08)	2.69 (2.33)	3.00 (2.39)
Theoretical range	–	12–15	0–24	0–24	0–24	0–24	0–18	0–18	0–10	0–10

*** Significant *p* value after applying the Bonferroni correction (45 comparisons, *p* < 0.001)

### Bi-Directional and Longitudinal Associations Between Parental Support and Parental Knowledge on Antisocial Behavior and Emotional Problems

Figure 1 shows the structural equation model predicting antisocial behavior and emotional problems. The model showed adequate values of goodness-of-fit indices, χ^2^ (863) = 1565.23, *p* < 0.001, χ^2^/DF = 1.81, CFI = 0.91, RMSEA = 0.04 [0.03, 0.04], SRMR = 0.06. Significant positive correlations (*p* < 0.001) were found between the latent variables Support T1-Knowledge T1 (*r* = 0.68), Support T1-Support T2 (*r* = 0.33), Knowledge T1-Knowledge T2 (*r* = 0.48), Antisocial behavior T3-Emotional problems T3 (*r* = 0.39), as well as between the observed control variables Antisocial behavior T1-Emotional problems T1 (*r* = 0.20). The correlation between the latent variables Support T2-Knowledge T2 (*r* = 0.05) was not significant (*p* = 0.227).

**Fig. 2:**
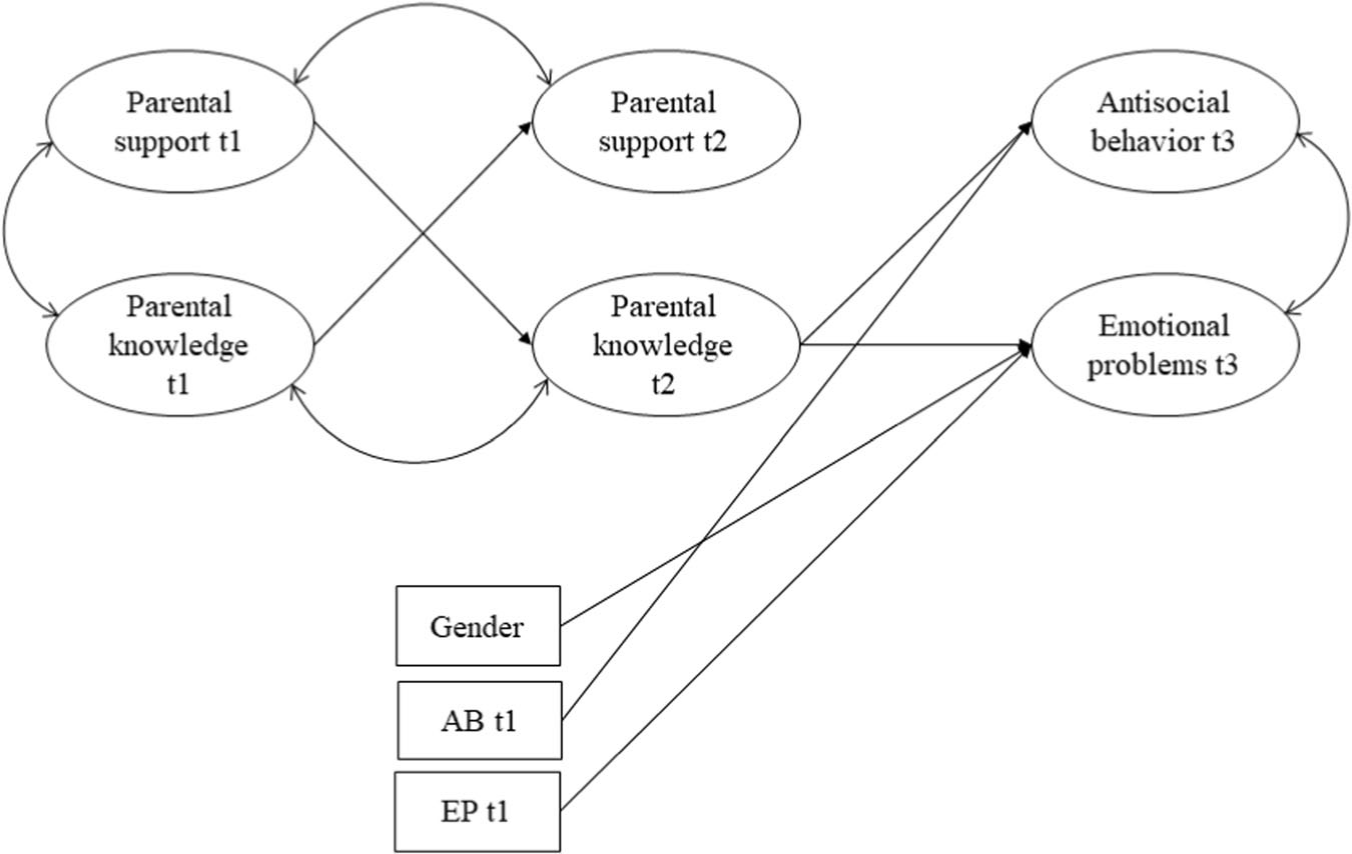
Significant Paths in the Structural Equation Model Tested. Non-significant paths are not shown. AB = Antisocial behavior. EP = Emotional problems

Table 2 displays standardized coefficients from the structural equation model and Fig. 2 shows the significant paths in the model. The results regarding the observed covariates indicated that gender significantly predicted emotional problems (females showed higher levels of emotional problems than males), T1 antisocial behavior significantly predicted antisocial behavior in T3 and T1 emotional problems significantly predicted emotional problems in T3, both in a positive direction. For the main variables of the model, results indicated that parental support and parental knowledge significantly predicted each other one year later, in a positive direction. Only parental knowledge in T2, not parental support, significantly and inversely predicted antisocial behavior and emotional problems in T3.

**Table 2:** Results of SEM Predicting Antisocial Behavior and Emotional Problems

	Parental support T2		Parental knowledge T2		Antisocial behavior T3		Emotional problems T3	
	B (SE)	β	B (SE)	β	B (SE)	β	B (SE)	β
Gender	−0.08 (0.05)	−0.07	0.04 (0.07)	0.04	−0.07 (0.05)	−0.07	0.23 (0.03)	0.32^***^
Age	0.01 (0.05)	0.01	−0.09 (0.05)	−0.12	−0.06 (0.06)	−0.07	0.02 (0.03)	0.04
AB T1					0.17 (0.07)	0.49^**^	−0.00 (0.02)	−0.00
EP T1					0.01 (0.02)	0.05	0.07 (0.01)	0.46^***^
Support T1			0.37 (0.08)	0.42^***^				
Knowledge T1	0.55 (0.07)	0.46^***^						
Support T2					0.10 (0.09)	0.10	−0.05 (0.06)	−0.08
Knowledge T2					−0.46 (0.20)	−0.41^***^	−0.15 (0.06)	−0.20^*^
R^2^	0.20		0.21		0.45		0.44	

*Note*. Gender was coded as 0-male, 1-female. AB = antisocial behavior. EP = emotional problems. Support = parental support. Knowledge = parental knowledge

^*^p < 0.05 ^**^p < 0.01 ^***^p < 0.001

### Mediating Role of Parental Knowledge in the Association Between Parental Support and Antisocial Behavior and Emotional Problems

To estimate the indirect effects, a combination of Maximum Likelihood (ML) and bootstrapping (*b* = 5000) were used in order to maximize accurate estimations. The results indicated significant indirect effects from parental support (T1) through parental knowledge (T2) on antisocial behavior (β = −0.17, *p* < 0.001; 95% CI −0.25, −0.10) and emotional problems (β = −0.08, *p* = 0.018; 95% CI −0.14, −0.03). The indirect effects from parental knowledge through parental support on antisocial behavior (β = 0.05, *p* = 0.181; 95% CI −0.01, 0.11) and emotional problems (β = −0.04, *p* = 0.263; 95% CI −0.09, 0.02) were not significant.

The sensitivity analysis testing the model with rule-breaking produced results that mirrored those reported above. The model showed very similar acceptable goodness-of-fit indexes, correlations and direct and indirect effects (both in direction and significance of coefficients). These results are available from the authors upon request.

## Discussion

Developmental theory suggests that warm and supportive parent-child relationships are the basis for later child disclosure and parental knowledge and research has confirmed that parental knowledge is one of the most robust predictors of adolescent adjustment. The mediating role of parental knowledge between parental support and adolescent outcomes has been mostly studied regarding antisocial behavior and externalizing problems compared to emotional problems, and there is a gap in research about assessing those effects on both behavioral and emotional problems in adolescence from a longitudinal perspective. From a developmental and holistic-integrative approach, the study of the longitudinal interrelation of the main positive parenting practices influencing co-occurring behavioral and emotional problems is warranted to better understand adolescent development.

To address these gaps, the current study analyzed bi-directional relationships and longitudinal mediation effects of parental support and parental knowledge on both antisocial behavior (externalizing problems) and emotional problems (internalizing problems) in Spanish normative adolescents. In addition, cross-sectional and longitudinal relationships between externalizing and internalizing problems were analyzed in order to shed further light on patterns of adjustment problems displayed by youth at this developmental stage. Although antisocial behavior did not predict emotional problems and emotional problems did not predict antisocial behavior two years later, antisocial behavior and emotional problems were significantly and positively correlated in T1 as well as in T3. These results confirm the co-occurrence of both externalizing and internalizing problems in adolescence (Loeber & Burke, 2011) and highlight the need to adapt prevention and intervention strategies accordingly (Ballester et al., 2020).

The findings indicated that parental support and parental knowledge were predictive of each other one year later, confirming the first hypothesis of the study. This finding is consistent with previous strong evidence indicating that parental support positively predicts parental knowledge (e.g., Liu et al., 2020; Yun et al., 2016). Evidence regarding the longitudinal effects of parental knowledge on parental support was not as consistent. Some studies have previously not found significant reciprocal, longitudinal relationships between parental acceptance and parental knowledge (Garthe et al., 2015), while others have found bi-directional connections between parental knowledge and positive parent-child relationships (Walters, 2019). The inconsistency regarding the effects of parental support might be due in part to the heterogeneity in the definition and, therefore, measure operationalization of the construct (e.g., support, warmth, acceptance, attachment) (Hoeve et al., 2009).

On the other hand, in the current study only parental knowledge at T2 was significantly related to antisocial behavior and emotional problems one year later. These findings confirm the role of parental knowledge in adolescence as a robust parenting-related predictor of both externalizing (Cutrín et al., 2019) and internalizing problems (Yap et al., 2014). Moreover, in line with other studies (Reitz et al., 2006), the effects of parental knowledge appeared to be stronger for externalizing than for internalizing problems in adolescence.

The results of this study did not show a direct influence of parental support on behavioral and emotional problems in mid-adolescence; hence, the second hypothesis was only partially supported. Previous research already identified the no significant direct effects of parental support on antisocial behavior (e.g., Cutrín et al., 2015) and emotional problems (e.g., Parra & Oliva, 2006). Nevertheless, parental support indirectly predicted both antisocial behavior and emotional problems two years later, through the level of parental knowledge.

As proposed in the third hypothesis, the significance and confidence intervals of indirect effects showed a unidirectionality from parental support through parental knowledge on adolescent adjustment, and not the reverse pathway. As other studies have found, the establishment of a supportive and communicative family atmosphere in early adolescence seems to promote parental knowledge during mid-adolescence, which, in turn, is protective against both antisocial behavior (e.g., Yun et al., 2016) and emotional problems (e.g., Yan et al., 2017). These findings support developmental theory and reinforce the evidence about parental support as the basis of later parental-adolescent relationships (Collins & Steinberg, 2008) and specifically of later parental knowledge, in line with previous research (e.g., Blodgett Salafia et al., 2009; Kerr et al., 2010; Tilton-Weaver, 2014).

These findings show that parental knowledge fully mediated the effects of parental support on externalizing and internalizing problems in Spanish adolescents. These findings confirm the results of previous cross-sectional research conducted in Spain regarding antisocial behavior (Cutrín et al., 2017) and provide novel evidence regarding the longitudinal effects of parental support through parental knowledge on emotional problems. Although parental support is not directly linked with externalizing and internalizing problems in adolescence, the results are indicative of the positive role of parental support in Spanish parenting styles (García & Gracia, 2010), as it is the key variable for later adolescent disclosure and, subsequently, parental knowledge, which is, in turn, the key for preventing both behavioral and emotional problems in adolescence.

Due to the existing regional cultural diversity within Spain, future intraregional studies could be of interest. Future cross-national research should also examine whether the influence of parental support on later communication patterns, willingness to disclose information, and parental knowledge (as suggested by previous research; e.g., Liu et al., 2020; Tilton-Weaver, 2014) is stronger in Spanish families than in families in other cultural contexts (e.g., in North Europe).

### Practical Implications

Prevention of adolescent behavioral and emotional problems should be approached as a public health issue and prioritized within communities and by social policy (Kim et al., 2015). Preventive strategies should be applied to avoid early engagement in antisocial behavior and manifestation of emotional problems, in order to promote a healthy, well-adjusted development throughout adolescence. Many preventive efforts so far have been aimed at the family as the main context for socialization and parenting practices have been a target of preventive interventions (e.g., *Familias: Preparando la Nueva Generación*, Marsiglia et al., 2014; *Programa de Competencia Familiar*, Orte et al., 2013; *Constuir Lo Cotidiano*, Torío et al., 2015).

The mediation effects found in the current study together with developmental theory (e.g., Granic et al., 2003) and previous research on parenting practices support the design and implementation of early prevention approaches. Interventions providing parental support can be protective for antisocial behavior and emotional problems in Spanish adolescents since it is a foundation of positive parent-child relationships later in life. Ultimately, the goal of preventive efforts to improve behavioral and emotional adjustment in adolescence should be to strengthen parental knowledge. In addition, positive parenting practices can promote the natural decline of antisocial behavior in the general population of adolescents (Buck & Dix, 2014). As this study was conducted with adolescents from the general population, it is important to highlight that parenting behaviors that might successfully prevent the onset of antisocial behavior (such as parental support; Zheng & Cleveland, 2013), might not reduce such behavior once it is established. Thus, the findings indicate the need to implement early and universal prevention interventions.

### Limitations

The current study presents some limitations that must be considered for the appropriate interpretation of the findings and should be addressed in future research. The use of only student self-reports can lead to shared method variance, which may have partially influenced the results. Parents did not participate in the study and their responses to the same questions answered by the youth could have enriched the findings. Future sample designs of research studies on this topic could benefit from parent-child dyads. In addition, students at higher risk were more likely to drop out of the study, which may have affected the generalizability of findings. The study only included school-enrolled students, having a sample that includes non-school attending youth would have provided a more accurate assessment of their experiences with delinquency. Additionally, it should be taken into account that levels of posttest attrition were significantly higher among students reporting more antisocial behavior at the pretest. Antisocial behavior is associated with poor school performance and early school dropout, as well as a pattern of defiance towards authority, all of which may have been reasons for attrition in this study. Finally, personality traits and differences across genders were not considered in the current study. Future longitudinal studies that address these limitations and begin at earlier developmental stages will be beneficial developing knowledge on how parenting practices influence children’s behavior from childhood to late adolescence.

## Conclusion

Developmental theory and previous research have proposed that parental support leads to higher parental knowledge, which, in turn, influences adolescent adjustment. These mediation effects have been confirmed mostly regarding antisocial behavior and externalizing problems, but there is a lack of evidence on emotional and internalizing problems. The current study addressed the longitudinal interrelation of parental support and parental knowledge, as the main positive parenting practices, and their potential effects on both behavioral and emotional problems. Because this study was based on a developmental and holistic-integrative approach, the findings could contribute to a better understanding of adolescent development by disentangling how parental support and parental knowledge are longitudinally interrelated and how complex their influence is on adolescent adjustment. The current study confirmed the co-occurrence of behavioral and emotional problems in Spanish adolescents and the bi-directional relationships between positive parenting practices. One of the main findings confirmed that supportive parenting practices involving warmth, closeness, and open communication indirectly prevent adolescents’ antisocial behaviors through increasing parental knowledge. The main novel finding was that the same longitudinal parenting relationships influenced adolescents’ internalizing problems such as anxiety and depression. These findings have specific implications for practice, prevention interventions, research and policy, not only for adolescents but also for their parents. This study highlights the role of parental knowledge as the key variable influencing antisocial behavior and emotional problems. Therefore, these findings can contribute to the development of strategies, at both behavioral and emotional levels, for adolescents and their parents, by indicating that increasing parental knowledge should be the target of educational-prevention programs.
